# Characterization of calcineurin from *Cryptococcus humicola* and the application of calcineurin in aluminum tolerance

**DOI:** 10.1186/s12896-017-0350-9

**Published:** 2017-03-29

**Authors:** Lei Zhang, Jing-jing Zhang, Shuai Liu, Hong-juan Nian, Li-mei Chen

**Affiliations:** Faculty of Life Science and Technology, Kunming University of Science and Technology, Kunming, China

**Keywords:** *Cryptococcus humicola*, Calcineurin, Calmodulin-binding domain, Transgenic yeast, Aluminum tolerance

## Abstract

**Background:**

Calcineurin (CaN) is a Ca^2+^- and calmodulin (CaM)-dependent serine/threonine phosphatase. Previous studies have found that CaN is involved in the regulation of the stress responses.

**Results:**

In this study, the growth of *Cryptococcus humicola* was inhibited by the CaN inhibitor tacrolimus (FK506) under aluminum (Al) stress. The expression of *CNA* encoding a catalytic subunit A (CNA) and its interaction with CaM were upregulated when the concentration of Al was increased. A CaM-binding domain and key amino acids responsible for interaction with CaM were identified. ∆CNAb with a deletion from S454 to A639 was detected to bind to CaM, while ∆CNAa with a deletion from R436 to A639 showed no binding to CaM. The binding affinities of CNA1 and CNA2, in which I439 or I443 were replaced by Ala, were decreased relative to wild-type CNA. The phosphatase activities of ∆CNAa, CNA1 and CNA2 were lower than the wild-type protein. These results suggest that the region between R436 and S454 is essential for the interaction with CaM and I439, I443 are key amino acids in this region. The ability of the *CNA* transgenic yeast to develop resistance to Al was significantly higher than that of control yeast. Residual Al in the *CNA* transgenic yeast culture media was significantly lower than the amount of Al originally added to the media or the residual Al remaining in the control yeast culture media. These findings suggest that *CNA* confers Al tolerance, and the mechanism of Al tolerance may involve absorption of active Al.

**Conclusions:**

Al stress up-regulated the expression of *CNA*. CaM-binding domain and key amino acids responsible for interaction with CaM were identified and both are required for phosphatase activities. *CNA* conferred yeast Al resistance indicating that the gene has a potential to improve Al-tolerance through gene engineering.

## Background

Ca^2+^ acts as one of the most diverse and widespread second messengers in cellular processes [1]. Calmodulin (CaM) is a small, ubiquitous protein that acts as a calcium sensor to mediate several Ca^2+^ signals [2]. CaM is assumed to be involved in the activation of many enzymes, including CaM-dependent protein kinases and protein phosphatase (calcineurin) [3–5]. Calcineurin (CaN) is a heterodimer consisting of a catalytic subunit A (CNA) and a regulatory subunit B (CNB) [6]. Although it is widely distributed, its primary sequence and structure is conserved among all eukaryotes. CNA contains a catalytic domain, a CNB-binding domain, a CaM-binding domain, and an autoinhibitory domain (AID) [7]. CaM binds to the CaM-binding domains on CaN and causes a conformational change that displaces an AID from the active site, which activates its phosphatase activity [8, 9].

CaM binding sequences on CNA are also called CaM recruitment signaling (CRS) motifs. A CaM target database (http://calcium.uhnres.utoronto.ca/ctdb) [10] has been built based on the structural and sequence information of the CRS motifs. This database can be used to predict whether a sequence contains a CaM-binding domain and to calculate the average hydrophobicity, the average hydrophobic moment, and the average propensity for a short peptide. Many Ca^2+^-dependent CRS motifs have been identified as 1–14 or 1–10 class [11]. Studies on these CaM-binding proteins revealed the possibility of interacting residues on CNA; however, there is no direct experimental evidence for the interaction.

Studies have shown that CaN play important roles in regulation of stress responses. In budding yeast (*Saccharomyces cerevisiae*), CaN is required to maintain cell viability during cation stress and pheromone-induced growth arrest [12–16]. Calcineurin-dependent genes are activated by heat and ionic stress as well as when the cell wall is destroyed [17–20]. Recent studies have established a role for CaN in responding to alkaline pH and endoplasmic reticulum (ER) stress [21, 22]. In *Candida albicans*, the main role of CaN is to respond to various stimuli or stress [23, 24]. Under normal growth conditions, CaN is not required for growth in *S. cerevisiae*. Ethanol stress can activate the calcium-mediated calcineurin/Crz1 pathway [25]. CaN play a role in the vitality of filamentous fungi, where a deletion mutant of *cna1* exhibited changes in growth efficiency, mycelium morphology and sporulation [26]. In *C. albicans*, a pathogen of human, mutants of the catalytic subunit of calcineurin (CMP1/CNA) and the calcineurin regulatory subunit (CNB1) are sensitive to salt stress, alkali stress and osmotic stress [27, 28].

Al exists in a trivalent cation (Al^3+^) form under acid conditions, which is a toxic to animals, plants and microbes. Al toxicity is one of the factors limiting crop productivity in acid soils. Studies about the mechanisms of Al tolerance can provide insight to solve the problem of Al toxicity. *Cryptococcus humicola* is a high Al-resistant yeast strain isolated from an acidic field [29]. In order to survive in acid soil, it has evolved Al-resistant mechanisms. Therefore, *C. humicola* can be used as a model for studying the mechanisms of Al toxicity and resistance. At the same time, the aluminum-resistant genes can be explored from *C. humicola* to improve the Al-tolerance of the crops. Our previous work proved that CaM signal pathway involve in response to Al stress. In *Rhodotorula glutinis*, an inhibitor of calcineurin completely inhibited the growth of under Al stress [30]. In this work, we aim to clone the gene of catalytic subunit of calcineurin (*CNA*) from *C. humicola*, analyze the expression of CNA under Al stress, identify the characteristics of interaction between CNA and CaM, and prove the Al-tolerance ability of this gene in yeast. These results provide experimental data for the interaction between CaM and CNA and a theoretical reference for application of the gene in Al-tolerance.

## Results

### The role of CNA in Al stress

**Fig. 1:**
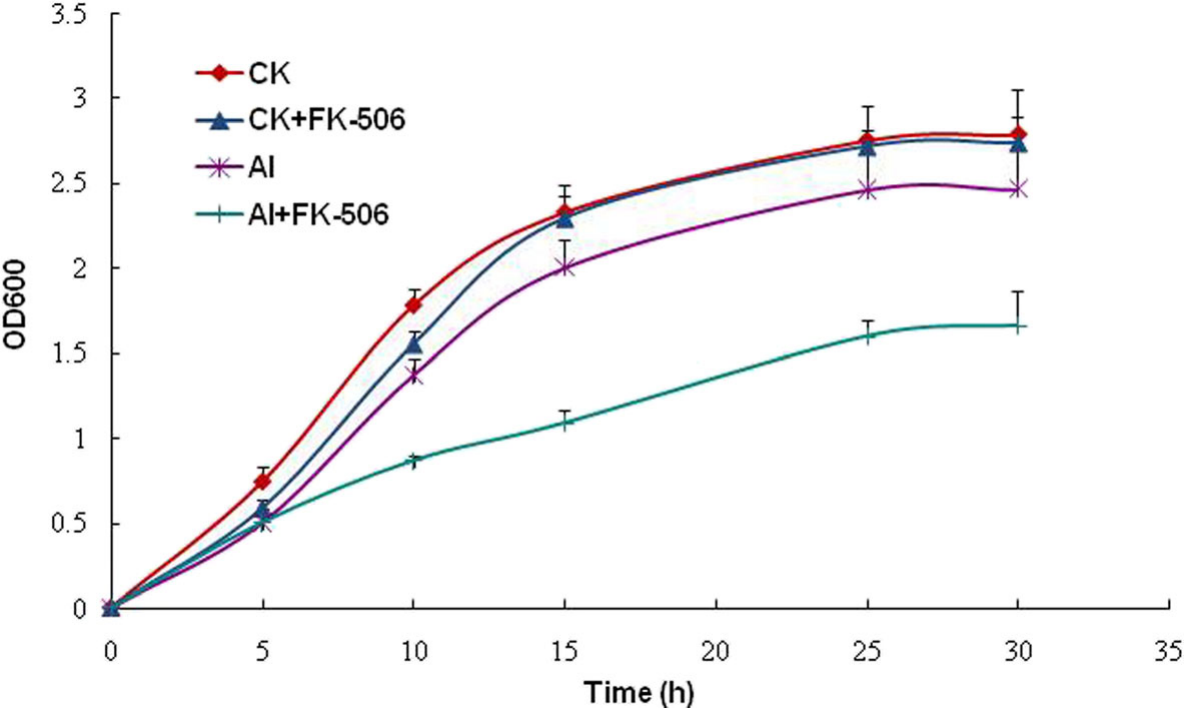
Effect of FK506 on the growth of *C. humicola* under Al stress. The initial OD_600_ of each culture was adjusted to 0.05, and FK506 was added to a final concentration of 1 μg/mL. The culture was then incubated at 30 °C while shaking at 200 rpm. The OD_600_ was measured every 2 h. FK506: Inhibitor of CaN. CK: Al-free medium; Al: Medium containing 50 mM Al^3+^; CK+ FK506: FK506 was added to the medium without Al; Al + FK506: FK506 was added to the medium containing 50 mM Al^3+^. Each sample was in triplicate, and three independent experiments were conducted

To examine the impact of CaN on the growth of *C. humicola* under Al stress, FK506 was added to the culture medium containing 50 mM Al^3+^. As shown in (Fig. 1), the addition of Al or FK506 in liquid medium without Al slightly inhibited the growth of the strain. However, when FK506 was added to culture medium containing Al, the growth of the strain was severely inhibited. These results suggest that CaN is involved in the growth of *C. humicola* under Al stress.

**Fig. 2:**
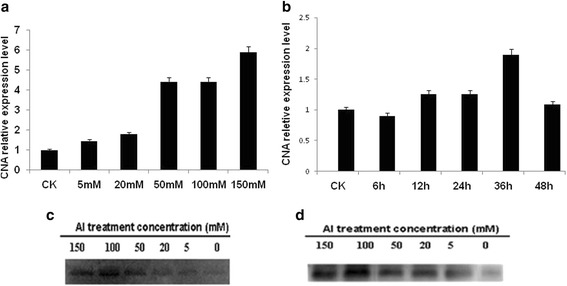
Analysis of the expression level of CNA (**a**, **b** and **c**) and interaction of CNA with CaM (**d**) under Al stress. qRT-PCR analysis of the transcription levels of *CNA* in the presence of different concentrations of Al (**a**) and different treatment time under 50 mM Al^3+^ (**b**). The results were expressed as relative values with respect to 0 mM or 0 h, which were set to 1.0, respectively. The data are presented as the mean ± SE (*n* = 3). **c** Expression analysis of CNA under Al stress. Total protein was extracted from cells treated with 5, 20, 50, 100, or 150 mM Al^3+^. The membranes were probed with mouse antibodies against CNA and with peroxidase-conjugated goat antibodies against mouse IgG. **d** The interaction level of CNA with CaM under Al stress. Purified GST-CaM and total protein from cells treated by Al were incubated together. GST agarose resin was added. The interaction was detected with anti-CNA and a specific polyclonal anti-CaM antibody

To study the transcription levels of *CNA* under Al stress, total RNA of cells treated with Al was used as the template for quantitative RT-PCR (qRT-PCR). As shown in (Fig. 2a and b) , the expression of *CNA* increased gradually in cells treated with increasing concentrations or with the extension of treatment time. The expression level reached the maximum amount (5.9-fold and 1.9-fold) when treated with 150 mM Al^3+^ or treated for 36 h. To further study the expression of CNA in translational levels under Al stress, cultures of *C. humicola* treated with Al were collected. Western blot analysis was used to analyze the impact of Al stress on CNA protein levels. As shown in Fig. 2c, the expression of CNA protein gradually increased as the Al concentration increased. When the concentration of Al was 100 mM, the expression of CNA reached its maximum level. These results indicate that Al stress can affect the translation of the CNA and that CNA is involved in the response to Al stress in *C. humicola*.

To validate the effect of Al stress on the interaction between CaM and CNA, GST-pull downs and Western blot analysis were used. As shown in Fig. 2d, the binding level of CNA and CaM upon treatment with increasing concentrations of Al showed a gradual upward trend and reached a maximum level at concentration of 100 mM Al^3+^, indicating that interaction between CaM and CNA was also affected by Al treatment.

### Prediction of the CaM-binding domain and the binding residues of CNA

**Fig. 3:**
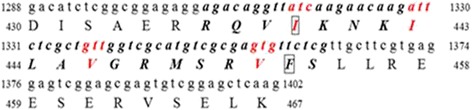
Partial nucleotide sequence of the cDNA for CNA and the deduced amino acids. The figures in the *left* and *right* margins indicate the amino acid and nucleotide positions, respectively. Italics represent the predicted CaM binding sequence. The amino acids shown in red indicate the position of hydrophobic residues that form the 1-8-14 motif. Anchor amino acids are indicated by *black boxes*

We submitted the CNA sequence (GenBank accession no. KJ738305) to the CaM target database (http://calcium.uhnres.utoronto.ca/ctdb) [10], which includes almost all published CRS motif and obtained the putative CaM-binding domain sequence. The cDNA of CNA encodes a 71.5-kDa protein with a CaM-binding domain in its C-terminal region. The conserved hydrophobic residues in the CaM-binding domain, I439, I443, V446, and V452, form a 1-8-14 motif, which is probably involved in the interaction with CaM. I439 and F453 were predicted to be the anchor amino acids that possibly play an essential role in binding with CaM. Therefore, we decided to identify the roles of these five amino acids, which are shown in (Fig. 3), in binding with CaM and their role in activation of the phosphatase.

### Expression and purification of the recombinant proteins

To determine the location of the CaM-binding domain in the CNA protein, a recombinant CNA protein of full length and two deletion mutants were expressed in *E. coli* as His-tagged fusion proteins. To identify the essential amino acids in the CaM-binding domain that bind to CaM, we constructed site-directed mutants of CNA. We mutated the five predicted amino acids to alanine. cDNA for CNA and all of the mutated CNAs were cloned into the pET-32a (+) vector, expressed in *E. coli* BL21 (DE3) cells, and then purified.

SDS-PAGE gels showed clear CaM recombinant protein bands at the position of the theoretical molecular weight of 42 kDa (including the protein tag weight of 26 kDa). Similarly, CNA and their site-directed mutant protein (CNA1, CNA2, CNA3, CNA4, and CNA5) were shown at the position of 89.5 kDa (including the protein tag weight of 18 kDa). The molecular weights of the truncated proteins ∆CNAb, ∆CNAa were 70.2 kDa and 67.3 kDa (including the protein tag weight of 18 kDa), respectively (data not shown).

### Far-western blot analysis

**Fig. 4:**
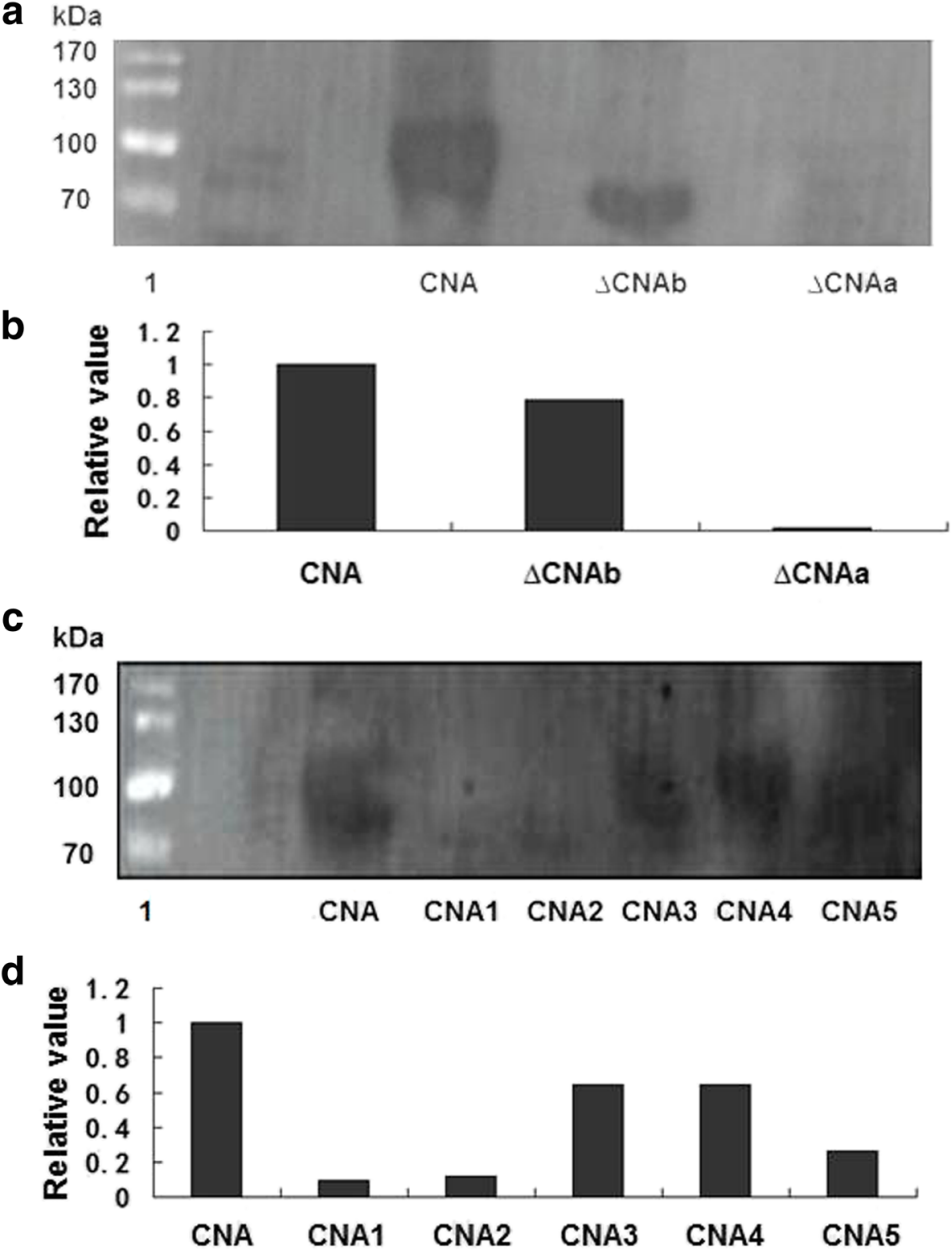
Analysis of the interaction between the recombinant proteins and CaM by Far-Western blot. **a** Far-Western blot analysis of the binding of CNA, ∆CNAb, ∆CNAa with CaM. Lane 1, protein standards; Proteins samples were 40 μg. **b** Analysis of the relative binding capacities of the truncated proteins with CaM. **c** Far-Western blot analysis of the binding of CNA, CNA1, CNA2, CNA3, CNA4, CNA5 with CaM. Lane 1, protein standards. Proteins samples were 20 μg. **d** Analysis of the relative binding capacities of the site-directed mutagenesis proteins with CaM. Relative activity analysis was performed by image J. The binding capacity of CNA with CaM was designated as 1

Binding of the truncated and mutant proteins to CaM were assayed using Far-Western blot analysis. To identify the CaM-binding domain, we transferred CNA and its truncated proteins to nitrocellulose and verified their binding characteristics to CaM. The deletion mutant ∆CNAb (with deletion from S454 to A639) was detected to bind to CaM, while the truncated protein ∆CNAa (with deletion from R436 to A639) showed hardly any binding with CaM (Fig. 4a and b). This result was expected because ∆CNAa lacks the putative CaM-binding region was detected hardly binding with CaM. The results indicated that the region between R436 and S454 was responsible for the interaction with CaM.

To determine which amino acid(s) in the CaM-binding domain was essential for the interaction with CaM, we mutated five conserved hydrophobic amino acids and found that all the five mutated proteins could bind with CaM (Fig. 4c). The binding capacity of CNA with CaM was stronger than that of the mutant proteins, while the binding capacity of CNA1 and CNA2 were the lowest of the five mutants (Fig. 4d). These results indicated that the conserved hydrophobic residues, I439, I443, V446, V452, and F453 all play a role in binding to CaM, while I439 and I443 are the most important amino acids involved in binding to CaM.

### The effect of the CaM-binding domain on the phosphatase activity of CNA

**Fig. 5:**
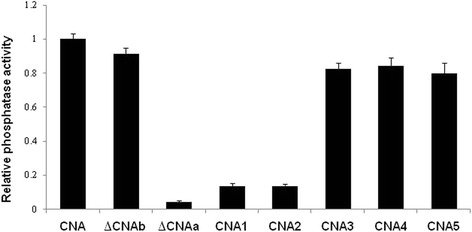
Comparison of the phosphatase activity of the mutant proteins with CNA. The molar ratio of CaM to CNA or its mutant proteins was 2:1. The absorbance was read at 410 nm. The activity of CNA was designated as 1. The experiments were repeated three times, and a representative set of data is shown

To determine the role of the CaM-binding domain in the activation of the phosphatase activity of CNA, the activities of CNA and its mutants were measured by a PNPP assay. The phosphatase activity of CNA was designated as 1. As shown in (Fig. 5), the activity of the truncated protein ∆CNAa was greatly decreased and proved to be the lowest among all of these mutant proteins. The phosphatase activity of CNA1 and CNA2 was also decreased and lower than the other three site-directed mutant proteins and the wild-type protein. These results suggest that the region from R436 to A639, Ile439 and Ile443 are not only important for binding to CaM but also for activation of the phosphatase activity.

### Al resistance of transgenic yeast

**Fig. 6:**
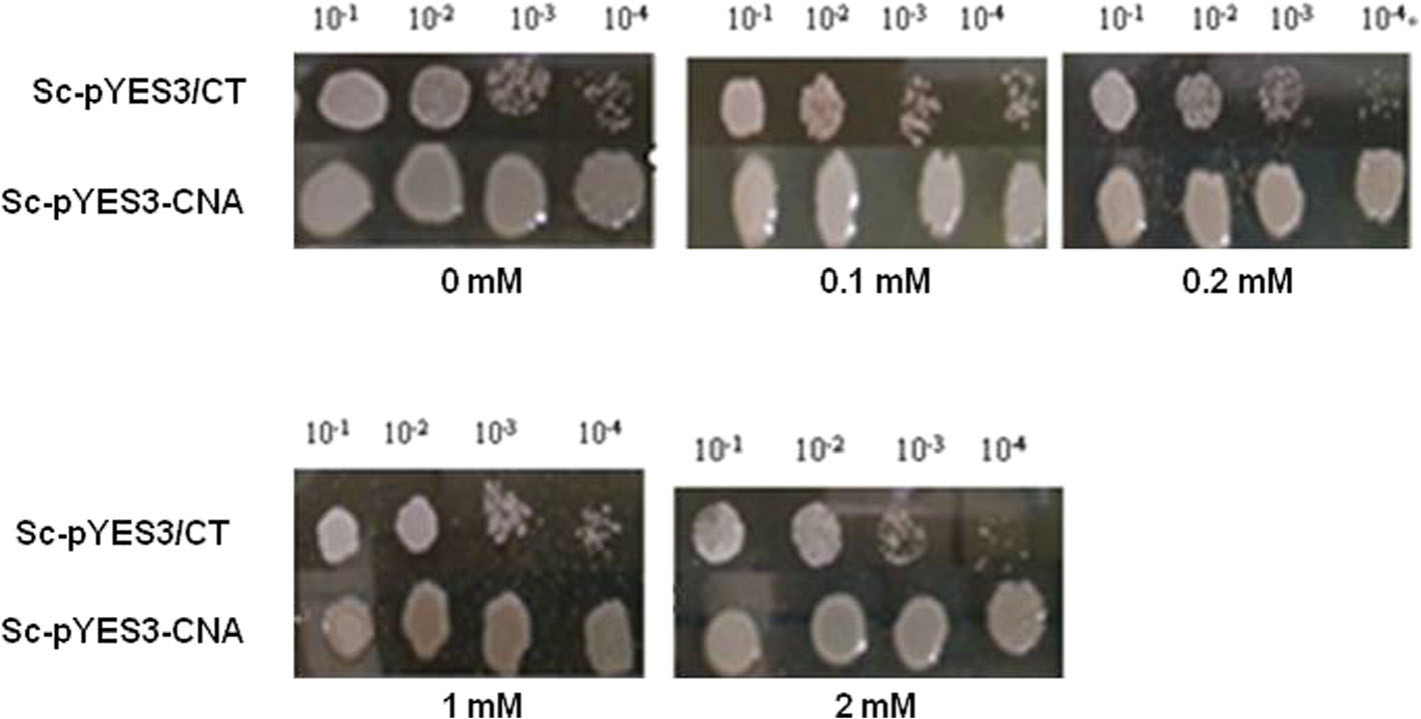
Growth of transgenic yeast on solid media under Al stress. Yeast cells were pre-incubated in SC-liquid medium containing 2% (w/v) glucose to an OD_600_ of 2.0. Ten-fold serial dilutions were prepared (1:10, 1:100, 1:1000 and 1:10,000), and 5 μL of each dilution was spotted onto SC-trp medium supplemented with 2% (w/v) galactose and 0 mM, 0.1 mM, 0.2 mM, 1 mM, or 2 mM Al^3+^. The results were recorded after the cultures were incubated at 30 °C for 3 days

**Fig. 7:**
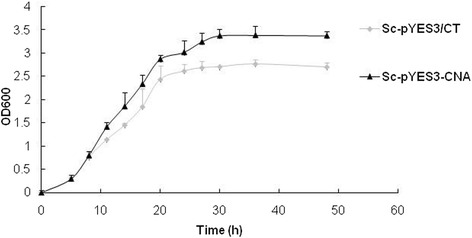
Al-tolerance analysis of transgenic yeast in liquid culture. The initial OD_600_ was adjusted to 0.05. The culture was then incubated at 30 °C while shaking at 200 rpm. The OD_600_ was measured every 2 h. Each sample was in triplicate, and three independent experiments were conducted. Vertical bars represent standard deviations of the means (*n* = 3)

To determine if *CNA* can confer Al tolerance in the transgenic yeast, a transgenic strain designated as Sc-pYES3-CNA was generated. The expression of the CNA protein in the transgenic yeast was verified by Western blot (data not shown). A control strain named Sc-pYES3/CT containing an empty vector and the transgenic strain were treated with different concentrations of Al. The results showed that the transgenic yeast grew better than the control strain on plates with or without Al. The transgenic yeast formed much bigger colonies than the control yeast when increasing the Al concentration. In the presence of different Al concentrations, the colonies formed by the transgenic strain were still much bigger than the colonies formed by the control strains (Fig. 6). The growth of transgenic yeast and control yeast was assayed in liquid broth with 2 mM Al^3+^. Within the first 20 h, there was no significant difference between the growth of control and transgenic strains. During the stationary phase, the transgenic yeast grew better than the control yeast (Fig. 7). These results suggest that *CNA* conferred Al tolerance in yeast.

### Detection of residual Al in culture

**Fig. 8:**
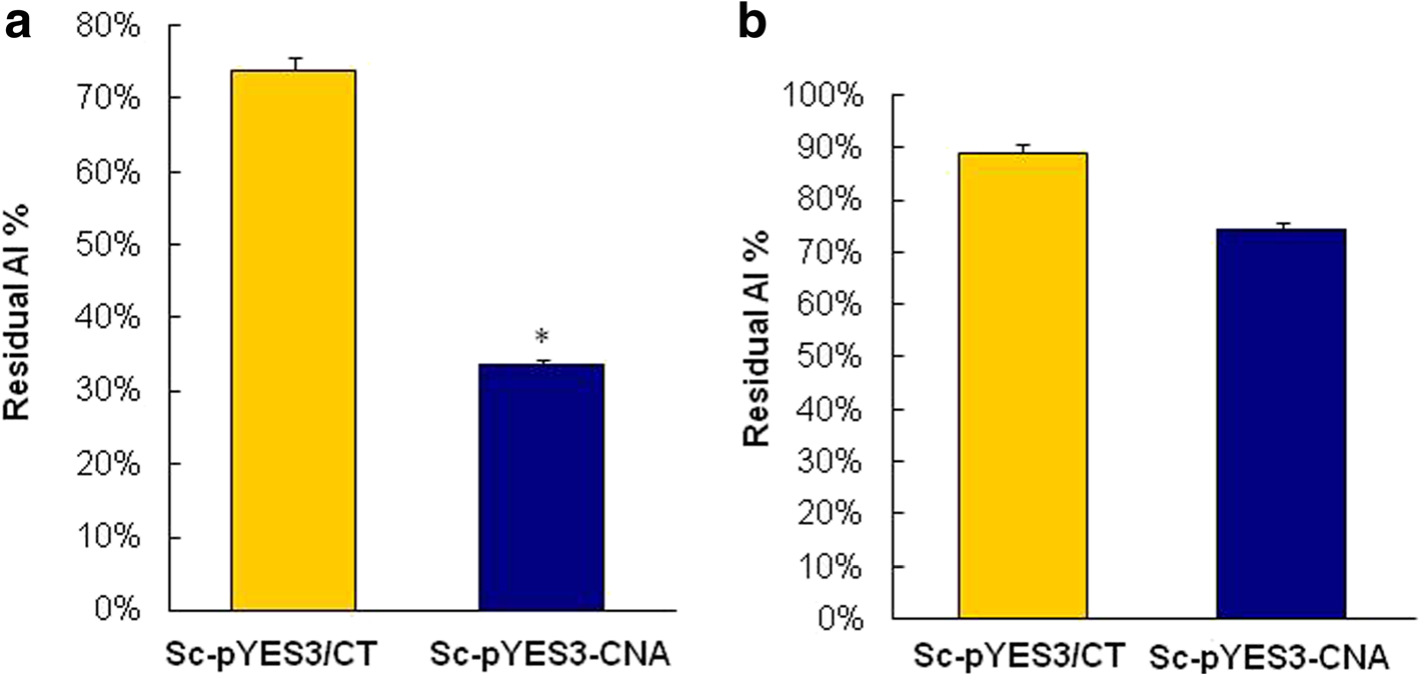
Assay of residual Al in the culture after incubation with transgenic yeast in the presence of 0.2 mM Al^3+^ (**a**) or 2 mM Al^3+^ (**b**). Uninoculated media was shaken and used as a negative control. The residual Al in the negative control was designated as 100%. All values are the means of three replicates. Asterisks represent significant differences when compared with the controls (*P* < 0.05)

To explore the reason for Al tolerance in the transgenic yeast, the residual levels of active Al in the culture after incubation of the yeast strains was determined. The transgenic yeast Sc-pYES3-CNA and the control yeast Sc-pYES3/CT were cultured in broth medium containing 0.2 mM and 2 mM Al^3+^. The residual Al levels in the supernatant were measured after eliminating the yeast cells. Uninoculated media was shaken and used as a negative control, and the residual Al content in this negative control was designated as 100%. The residual Al content in the medium containing 0.2 mM Al^3+^ and the transgenic yeast in culture was significantly reduced to 33.54% of the initial content (Fig. 8a). The residual Al content in the medium containing 2 mM Al^3+^ with the transgenic yeast in culture was also significantly reduced to 74.19% of the initial content (Fig. 8b). The residual Al in culture of control yeast had a slight reduction probably due to weak absorption of the cells. Compared with the control strain, 40.36 and 14.78% of the initial Al content was removed by the transgenic yeasts when cultured in medium containing 0.2 mM and 2 mM Al^3+^, respectively. We speculated that the transfer of *CNA* might promote the yeast to absorb the active Al from the culture. The adsorption of Al onto the cell surface or on the inside of the cells will need to be investigated further.

## Discussion

Several CaM-binding proteins, such as neuromodulin, myosin and calcineurin, have been identified in mammalian tissues. In this study, we characterized calcineurin from *C. humicola*. Yap et al. [11] had built a CaM target database (http://calcium.uhnres.utoronto.ca/ctdb) [10] based on CRS motifs structural and sequence information. In the present study, we identified a putative CaM-binding domain in CaN and five key amino acids responsible for the interaction between CaN and CaM based on the database.

Our predicted result showed that conserved hydrophobic residues in the CaM-binding domain formed 1-8-14 motif. Because alanine has a relatively stable conformation and has little impact on the properties of the protein [31], we substituted alanine for each of these five amino acids in the CaM-binding domain. We found that these five amino acids were associated with CaM binding, and I439 and I443 were identified as the most important amino acids involved in binding to CaM. The positions of these five hydrophobic residues in the CaM-binding domain are similar to their counterparts in short peptides. I396, I400, I403, V409, and F410 play an important role in binding with CaM [32]. These results provide insight into the interactions between CaM and CaM-binding proteins.

Several studies have demonstrated that signal pathways are involved in Al tolerance in *S. cerevisiae* and *R. glutinis*, including the SLT2 mitogen-activated protein kinase signal transduction pathway, the calmodulin-dependent protein phosphatase (calcineurin) signaling pathways and the calmodulin-dependent protein kinase [30, 33, 34]. Cyclosporin A is an inhibitor of calcineurin. The addition of cyclosporin A completely inhibited the growth of *R. glutinis* under Al stress. Our work demonstrated how the gene encoding calcineurin is involved in response to the aluminum stress and resistance to aluminum toxicity.

Previous studies have demonstrated that cell walls contributed to metal ion tolerance in fungi, such as Cd, Zn, Ni, Cu and Hg resistance [35–39]. Al stress also led to the increased thickness of the cell walls in *Rhodotorula* sp. RS1. The thickened cell walls contained 78% of the intracellular Al [40]. Calcineurin is required for cell wall biosynthesis. A variety of cell wall synthetic genes, such as genes encoding chitin synthase type I, 1,3-β-glucan synthesis regulator *RHO1* and 1,3-β-glucan synthase are regulated by the calcineurin/Crz1 signal pathway [41]. In *C. albicans*, calcineurin is also important for FKS1, the β-1,3 glucan synthase subunit [42]. Expression of *FKS1* encoding a component of the β-1,3-glucan synthase complex was increased in the *C. neoformans cnb1* mutant [43]. Fortwendel et al. reported that calcineurin signaling regulated chitin synthases at the transcriptional level [44]. The cell wall was thicker in all of the calcineurin deletion mutants of *A. fumigatus* [45]. In this study, residual Al in the medium containing CNA transgenic yeast in culture was significantly reduced compared to the original Al content in the medium. Therefore, we speculate that Al may be absorbed by the cell walls of the transgenic yeast. Whether CNA increases Al resistance through cell wall biosynthesis is currently being studied in our laboratory.

## Conclusions

In this study, FK506 inhibited the growth of *C. humicola* and the expression of *CNA* was upregulated under Al stress indicating that CNA involved in Al response. We used deletion and mutagenesis method to prove that the region between R436 and S454 is essential for the interaction with CaM and I439, I443 are key amino acids in this region responsible for interaction with CaM. When introduced into yeast, *CNA* could confer resistance to Al. The possible mechanism of Al tolerance is absorption of active Al.

## Methods

### Strains and culture conditions

*C. humicola* BSLL1-1 is an Al-resistant yeast that was isolated from acidic soil [29]. *C. humicola* cells were cultured at 30 °C in GM medium with pH 3.0 (glucose, 1.0%; peptone, 0.05%; yeast extract, 0.02% and MgSO_4_ · 7H_2_O, 0.02%) and supplemented with different concentrations of Al^3+^ when necessary [46]. *Escherichia coli* Trans1-T1 (TransGen Biotech, Beijing, China) was used for the genetic cloning. The pET-32a (+) and pGEX-4 T-1 plasmids were used for construction of expression vectors. *E. coli BL21 (DE3)* (Invitrogen, USA) was used for recombinant protein expression. The yeast expression vector pYES3/CT and *S. cerevisiae* strain INVSc1 (*MATa his3D1 leu2 trp1-289 ura3-52*) were used to generate transgenic yeast (Invitrogen, USA).

### Growth assays with addition of FK506

Cells were cultured until they reached an OD_600_ of approximately 1.0. The initial OD_600_ was adjusted to 0.05, and then the inhibitor of CaN tacrolimus (FK506) was added to the culture at a final concentration of 1 μg/mL. The cells were then incubated at 30 °C while shaking at 200 rpm. The OD_600_ of the cultures was measured every 2 h using a spectrophotometer (SHIMADZU, UV-1700, Japan). Each sample was tested in triplicate, and three independent experiments were conducted.

### Expression analysis of CNA at transcriptional and translational level

To detect the expression of the CNA gene at transcriptional level, qRT-PCR was conducted. RNA was extracted from cells treated with 5, 20, 50, 100, or 150 mM Al^3+^ for 12 h and cells treated with 50 mM Al^3+^ for 12 h, 24 h, 36 h, or 48 h using RNAiso Plus (TaKaRa, Dalian). PrimeScript ^TM^ RT reagent Kit was used for synthesizing the first strand cDNA (TaKaRa, Dalian) according to the manufacturer’s instructions. The cDNA was used as a template for PCR amplification of the CNA gene with a specific primer pairs CNA-qRT-F and CNA-qRT-R (Table 1). 18S rDNA amplified with the 18S-F and 18S-R primers (Table 1) was used as an internal control. Each sample was tested in triplicate.

**Table 1:** All primers used in this study

Primer pairs	Description	Forward primer	Reverse primer
CNA-qRT-F and CNA-qRT-R	Partial fragment of CNA gene was 190 bp	AACGGTGCGGAGGGTAT	GCTGTGCCAGTCTTGTCG
18S-F and 18S-R	Partial fragment of 18S rDNA gene was 198 bp	ATGCTGAAAAGCCCCGACT	ATTCCCCGTTACCCGTTG
CaM-F and CaM-R	the fragment of CaM gene was 450 bp	5′-GAATTCATGGCGGAGCAGCTGACCAAG-3′(*Eco*RI site underlined)	5′-CTCGAGTTACTTGGCCATCATCATG GTAAC-3′ (*Xho*I site underlined)
CNA-F and CNA-R	the fragment of CNA gene was 1917 bp	5′-GAATTCATGACCTC TCCGGCGAC-3′ (*Eco*RI site underlined)	5′-*GCGGCCGC*TTAGGCAATAGAGTTC TC-3′ (*Not* I site underlined)
CNAa-F and CNAa-R	1-1284 bp fragment of CNA, excluding calmodulin binding domain	5′-GAATTCATGACCTCTCCGGCGAC-3′ (*Eco*RI site underlined)	5′-*GCGGCCGC*CCTC TCCGCCGAGATGTC -3′ (*Not* I site underlined)
CNAb-F and CNAb-R	1-1362 bp fragment of CNA, including calmodulin binding domain	5′-GAATTCATGACCTCTCCGGCGAC-3′ (*Eco*RI site underlined)	5′-GCGGCCGCCGAGAACACTCGCGACAT-3′ (*Not* I site underlined)
mut-∆CNA1-F and mut-∆CNA1-R	Replace Ile439 with Ala439	GGAGAGGAGACAGGTTGCCAAGAACAAGATTCTC	GAGAATCTTGTTCTTGGCAACCTGTCTCCTCTCC
mut-∆CNA2-F and mut-∆CNA2-R	Replace Ile443 with Ala443	GGTTATCAAGAACAAGGCTCTCGCTGTTGGTCGC	GCGACCAACAGCGAGAGCCTTGTTCTTGATAACC
mut-∆CNA3-F and mut-∆CNA3-R	Replace Val446 with Ala446	AACAAGATTCTCGCTGCTGGTCGCATGTCGCGA	TCGCGACATGCGACCAGCAGCGAGA ATCTTGTT
mut-∆CNA4-F and mut-∆CNA4-R	Replace Val452 with Ala452	GGTCGCATGTCGCGAGCGTTCTCGTTGCTTCGT	ACGAAGCAACGAGAACGCTCGCGACATGCGACC
mut-∆CNA5-F and mut-∆CNA5-R	Replace Phe453 with Ala453	CGCATGTCGCGAGTGGCCTCGTTGC TTCGTGAG	CTCACGAAGCAACGAGGCCACTCGC GACATGCG

To detect the expression level of CNA protein under Al stress, the protein samples were prepared from cells treated with 5, 20, 50, 100, or 150 mM Al^3+^ for 12 h using a Total Protein Extraction kit (Sigma). 500 μg of total protein was separated using SDS-PAGE (12%) and then transferred to PVDF-P membranes. The membranes were probed for mouse antibodies against CNA with peroxidase-conjugated goat antibodies against mouse IgG. The antibodies were obtained using the polypeptides of CNA (RLAEVISSPTKGGQGER).

### GST-pull down and Western blot analysis

Purified glutathione S-transferase tagged CaM protein (GST-CaM) was used to study the interaction between CaM and CNA. 50 μg of purified GST-CaM and 500 μg of total protein from cells treated by 5, 20, 50, 100, 150 mM Al^3+^ for 12 h were added into binding buffer (50 mM Tris-Cl, pH 7.5, 100 mM NaCl, 0.25% Triton X-100, 1 mM EDTA, and 1 mM DTT) and then incubated at room temperature for 2 h. 30 μL GST agarose resin was added into the above mixture to bind overnight while shaking at 4 °C (40 rpm/min). The precipitate was collected by centrifugation at 4 °C, 3500 × g for 5 min, and then washed three times using the binding buffer. 10 μL SDS gel loading buffer (Tris–HCl 50 mM, pH 6.8; SDS 2%; DTT 100 mM; 0.1% bromophenol blue; and 10% glycerol) was added to the sedimented protein mixture, and the mixtures were boiled in boiling water bath for 10 min. The proteins were separated on a 12% SDS -PAGE gel. The separated proteins were transferred to PVDF membrane, and then incubated with anti-CNA antibodies and a specific polyclonal anti-CaM antibody. The results were imaged using a Chemidoc XRS (BIO-RAD). The antibodies against CaM were prepared with purified CaM proteins as described below.

### Construction of expression vectors

Total RNA was isolated from *C. humicola* by using Trizol reagent (Invitrogen, USA) according to the manufacturer’s instructions. Then, DNase I (Promega, USA) was used to remove residual genomic DNA contamination in the RNA. The quality of the RNA was examined by gel electrophoresis. cDNA was synthesized using M-MLV reverse transcriptase (Fermentas, Lithuania).

Gene fragments of CaM and CNA were generated by PCR amplification. The primers were listed in Table 1. Both fragments were cloned into a pMD-18 T vector (TaKaRa, Japan) to generate pMD-18 T-CaM and pMD-18 T-CNA. These plasmids were used for gene sequencing to verify their sequence. The CaM encoding gene was obtained from pMD-18 T-CaM digested with *Eco*RI and *Xho*I. The CNA encoding gene was obtained by digesting pMD-18 T-CNA with the *Eco*RI and *Not*I. The CaM fragment was then ligated into the pGEX-4 T-1 vector between the *Eco*RI and *Xho*I sites to generate pGEX-4 T-1-CaM.

To generate truncated constructions of CNA, ∆CNAa and ∆CNAb containing the 1–1284 bp and 1–1362 bp fragments of CNA were amplified using the primer pairs CNAa-F and CNAa-R, and CNAb-F and CNAb-R, respectively. ∆CNAa and ∆CNAb were cloned into pET-32a (+) to produce pET-32a-∆CNAa and pET-32a-∆CNAb, respectively. The difference between the two deletion mutants is whether or not they include the putative CaM binding domain.

For site-directed mutagenesis, mutations were generated using a QuikChange site-directed mutagenesis kit (TransGen, Beijing, China) according to the manufacturer’s instructions. We replaced five amino acids that were predicted to interact to CaM with alanine according to the literature [47] (I439 to A439, I443 to A443, V446 to A446, V452 to A452, and F453 to A453). The corresponding fragments were designated as CNA1, CNA2, CNA3, CNA4 and CNA5, respectively. Site-directed mutagenesis of these residues was performed using the mutagenic primers listed in Table 1. The mutated sequences were verified by sequence analysis. The CNA, CNA1, CNA2, CNA3, CNA4, and CNA5 fragments were ligated into the pET-32a(+) vector between the *Eco*RI and *Not* I sites to generate pET-32a-CNA, pET-32a-CNA1, pET-32a-CNA2, pET-32a-CNA3, pET-32a- CNA4, and pET-32a-CNA5.

### Expression and purification of CaM and CNA mutant proteins

These prokaryotic expression vectors were transformed into *E. coli* BL21 (DE3). For CaM recombinant protein expression, cells were grown at 37 °C in LB medium (10 g/L tryptone, 5 g/L yeast extract, 10 g/L NaCl) with 100 μg/mL ampicillin for 2 h; then, 1 mM IPTG was added. After growing for an additional 8 h at 37 °C with shaking at 80 rpm, cells were collected by centrifugation (3000 × g for 5 min). Cell pellets were resuspended in the cold PBS buffer (137 mM NaCl, 2.7 mM KCl, 10 mM Na_2_HPO_4_, 2 mM KH_2_PO_4_). The cell suspension was sonicated on ice for 7 min and then centrifuged at 10,000 × g for 15 min at 4 °C. The supernatant and the precipitate were subjected to SDS-PAGE. Proteins were visualized by staining with Coomassie blue. CaM recombinant protein was purified from the supernatant using glutathione-agarose beads according to the manufacturer’s instructions (Cwbiotech, Beijing, China). Rabbit was immunized with the purified CaM recombinant protein, and the anti-CaM antibodies were prepared from the immunized rabbit serum and used for Western analysis.

For CNA and CNA variant proteins expression, cells were grown at 37 °C in LB medium with 100 μg/mL ampicillin for 2 h; then, 1 mM IPTG was added. After growing for an additional 10 h at 20 °C with shaking at 80 rpm, the cells were collected by centrifugation (3000 × g for 5 min). The cell pellets were resuspended in the cold PBS buffer. The cell suspension was sonicated on ice for 7 min and then centrifuged at 10,000 × g for 15 min at 4 °C. Analysis of protein expression was performed in SDS-PAGE gel. His-tagged recombinant proteins were purified by affinity chromatography.

### Far-western analysis

For Far-western analysis, CNA and its variant proteins were subjected to SDS-PAGE and then transferred to nitrocellulose membranes. Membranes were denatured by incubating them in 6 M, 3 M, 1 M guanidine hydrochloride AC buffer (10% Glycerol, 0.1 M NaCl, 20 mM Tris–HCl, pH 7.5, 1 mM EDTA, 0.1% Tween-20, 2% Milk powder, 1 mM DTT) for 2 h at room temperature with shaking at 50 rpm and then renatured by incubating them in AC buffer without guanidine hydrochloride for 8 h at 4 °C while shaking at 50 rpm. After these steps, the membranes were blocked for 2 h at room temperature in TBST buffer (50 mM Tris–HCl, pH 8.0, 0.15 M NaCl, 0.02% Tween-20) containing 5% milk, rinsed with distilled water, and then incubated with 1 μg/mL CaM in binding buffer (50 mM Tris–HCl, pH 7.5, 1 mM EDTA, 100 mM NaCl, 1 mM DTT, 0.25% Triton X-100) containing 5 mM Ca^2+^ for 6 h at 4 °C with shaking at 50 rpm. Anti-CaM antibodies were added to binding buffer for another 6 h with the same conditions to the binding step. Membranes were washed three times with TBST buffer, and then incubated for 2 h in TBST with secondary antibody IgG-HRP (Cwbiotech, Beijing, China). CaM was bound by a CaM antibody, and then the CaM antibody was detected with horseradish peroxidase-conjugated secondary antibody and visualized using ECL reagent (Cwbiotech, Beijing, China).

### Assay of phosphatase activity

The phosphatase activity of CNA was assayed using *p*-nitrophenyl (pNPP) substrate solution. The reaction solution contained 50 mM Tris–HCl (pH 7.4), 0.5 mM DTT, 0.5 mM MnCl_2_, 1 mM CaCl_2_, 0.2 mg/mL bovine serum albumin, and 20 mM *p*-nitrophenyl phosphate. The reactions were performed in a volume of 0.2 mL at 30 °C for 20 min and terminated by the addition of 1.8 mL of a 0.5 mM Na_2_CO_3_ and 20 mM EDTA solution. The molar ratio of CaM to CNA or its mutants was 2:1. The absorbance was read at 410 nm.

### Construction of the yeast expression vector of *CNA* and transgenic yeast

To analyze the role of CNA gene in Al tolerance, the pYES3/CT yeast expression vector and the *S. cerevisiae* strain INVSc1 (*MATa his3D1 leu2 trp1-289 ura3-52*) were used to generate transgenic yeast. The coding region of CNA was digested from pMD-CNA with *Eco*RI/*Xho*I, and the fragments were purified and cloned into the *Eco*RI/*Xho*I site of pYES3/CT, which carries the *TRP1* selection marker. CNA expression was controlled by the inducible GAL1 promoter. Sequence analysis was used to confirm the insertion of *CNA* into the pYES3/CT. The resulting pYES3-CNA and pYES3/CT empty vector were transformed into the INVSc1 *S. cerevisiae* strain using the lithium acetate method. The presence of the pYES3-CNA plasmid in transgenic yeast was confirmed by PCR analysis and by Western blot using CNA antibodies.

### Al tolerance analysis of CNA in *S. cerevisiae*

The INVSc1 strain was inoculated from colonies on yeast extract peptone dextrose (YPD) medium plates (yeast extract 10 g, peptone 20 g, glucose 20 g, agar 20 g, and 1,000 mL water) and grown for 3 days at 30 °C. The transformants were grown on selective culture medium that lacked tryptophan (SC-trp: 0.67% yeast nitrogen base without amino acids but containing ammonium sulfate; 2% glucose; 0.01% (adenine, arginine, cysteine, leucine, lysine, threonine and uracil); 0.005% (aspartic acid, histidine, isoleucine, methionine, phenylalanine, proline, serine, tyrosine, and valine); and 2% agar) for 2 days at 30 °C, and the resulting trp^+^ colonies were selected for further research.

For the Al tolerance assays, yeast cells harboring pYES3-CNA and the empty vector pYES3/CT (control) were pre-incubated in SC-liquid medium containing 2% (w/v) glucose at 30 °C until they reached an OD_600_ of approximately 2.0. Then, 10-fold serial dilutions were prepared (1:10, 1:100, 1:1000 and 1:10,000), and 5 μL of each dilution was spotted onto SC-trp medium supplemented with 2% (w/v) galactose and 0 mM, 0.1 mM, 0.2 mM, 1 mM, or 2 mM Al^3+^. The cultures were incubated at 30 °C for 3 days. Each sample was spotted in triplicate, and three independent experiments were conducted. The INVSc1 strain containing pYES3/CT was used as a control. To assay the growth in liquid broth supplemented with 2 mM Al^3+^, the initial OD_600_ was adjusted to 0.05 and then the yeasts were incubated at 30 °C while shaking at 200 rpm. The OD_600_ of the cultures was measured every 2 h using a spectrophotometer (SHIMADZU, UV-1700, Japan).

### Residual Al content assays

Yeast cells harboring pYES3/CT-CNA and pYES3/CT were incubated overnight in SC-liquid medium containing 2% (w/v) glucose and were then transferred to YPD medium containing 0.2 mM or 2 mM Al^3+^ with an initial OD_600_ of 0.1. After 24 h of incubation, the culture was centrifuged at 12,000 rpm for 10 min, and the supernatant was filtered using a sterilized filter with 0.2 μm pores. The levels of inorganic monomeric Al and total Al in the filtered supernatant were determined according to the method described by Nian et al. [29]. The analyses were performed in duplicate.

## Abbreviations

AID: autoinhibitory domain
Al: aluminum
CaN: calcineurin
CaM: calmodulin
CNA: catalytic subunit A
CNB: regulatory subunit B
CRS: CaM recruitment signaling
ER: endoplasmic reticulum
SC-trp: selective culture medium lacked tryptophan
YPD: yeast extract peptone dextrose
